# Cromoglycate and nedocromil enhanced the reactive oxygen species-dependent suppressions with, but not without, dexamethasone in ischaemic and histamine paw oedema of mice

**DOI:** 10.1080/09629359791514

**Published:** 1997-12

**Authors:** Y. Oyanagui

**Affiliations:** 1 2nd Pharmacology Drug Development Laboratories I Fujisawa Pharmaceutical Co. 2-1-6 Kashima, Yodogawa-ku Osaka 532 Japan; 2 Redox Research Laboratory 97-1 Ishizuka Gokasho-cho Kanzaki-gun Siga-ken 529-14 Japan

## Abstract

Anti-inflammatory actions of two anti-allergic drugs, alone or with dexamethasone (Dex) were examined in two models, because inflammation is claimed to be important for allergic events, especially for asthma. Cromoglycate and nedocromil were tested in ischaemic- and histamineinduced paw oedema models of mice. These antiallergic drugs (1–100 mg/kg, i.p.) failed to suppress these oedemata, but enhanced the suppressions by a low dose of dexamethasone (0.1 mg/kg, s.c.) at 3–8 h after Dex injection. The mode of effects by anti-allergic drugs resembled that of a natural antioxidant (α-tocopherol, β-carotene etc.), and was different from that of an immunosuppressant like FK506. The enhancing potencies of the two anti-allergic drugs were similar at 6 h after Dex in both oedemata, and were diminished by superoxide dismutase (SOD) or catalase (i.p.). Cycloheximide completely abolished suppressions. Nedocromil, but not cromoglycate, inhibits inflammatory events. Therefore, there are common unknown actions by which the two anti-allergics enhance suppression by Dex. A possible mechanism of this action was supposed to enhance the superoxide and/or hydrogen peroxide-dependent glucocorticoid receptor (GR) signalling in the target cells.


Mediators of Inflammation, 6, 369–374 (1997)

### Cromoglycate and nedocromil enhanced the reactive oxygen species-dependent suppressions with, but not without, dexamethasone in ischaemic and histamine paw oedema of mice

Y. Oyanagui

2nd Pharmacology, Drug Development Laboratories I, Fujisawa Pharmaceutical Co., 2-1-6 Kashima, Yodogawa-ku, Osaka, 532, Japan
Tel: (+81) 6 390 1156
Fax: (+81) 6 304 5367

Present address: Redox Research Laboratory, 97-1 Ishizuka, Gokasho-cho, Kanzaki-gun, Siga-ken, 529-14, Japan

**Anti-inflammatory actions of two anti-allergic drugs, alone or with dexamethasone (Dex) were examined in two models, because inflammation is claimed to be important for allergic events, especially for asthma. Cromoglycate and nedocromil were tested in ischaemic- and histamine-induced paw oedema models of mice. These anti-allergic drugs (1–100 mg/kg, i.p.) failed to suppress these oedemata, but enhanced the suppressions by a low dose of dexamethasone (0.1 mg/kg, s.c.) at 3–8 h after Dex injection. The mode of effects by anti-allergic drugs resembled that of a natural antioxidant (α-tocopherol, β-carotene etc.), and was different from that of an immunosuppressant like FK506. The enhancing potencies of the two anti-allergic drugs were similar at 6 h after Dex in both oedemata, and were diminished by superoxide dismutase (SOD) or catalase (i.p.). Cycloheximide completely abolished suppressions. Nedocromil, but not cromoglycate, inhibits inflammatory events. Therefore, there are common unknown actions by which the two anti-allergics enhance suppression by Dex. A possible mechanism of this action was supposed to enhance the superoxide and/or hydrogen peroxide-dependent glucocorticoid receptor (GR) signalling in the target cells.**

**Key words:** Cromoglycate, Nedocromil, Dexamethasone, Ischaemic paw oedema, Histamine paw oedema, Asthma, Superoxide dismutase, Catalase, Cycloheximide

### Introduction

The effect of glucocorticoid (GC) appears in most models several hours after its dosing, because GC needs a lag time to induce the directly acting anti-inflammatory or anti-ischaemic proteins such as macrocortin (recently referred to as lipocortin)[1] and vasoregulin.[2] Typical GC, dexamethasone (Dex) or cortisol did not suppress ischaemic and histamine paw oedema of mice at 1 h after injection and showed a suppression peak at 3 h. However, when mice were pretreated by an immunosuppressant like FK506 (0.01–1 mg/kg, oral), suppression appeared earlier, at 30 min and 1 h after Dex. FK506 increased the suppression at 3 h, but not at 6 h after Dex injection.[3] Pretreatment by a natural antioxidant (α-tocopherol, β-carotene etc.) did not suppress the paw oedemata at 1 h after Dex, and enhanced the suppression at 3–18 h after Dex. $ED_{30}$ of them (i.p.) at 6 h after Dex was from 0.02 (morin) to 12 mg/kg (ascorbate) when 0.1 mg/kg Dex alone suppressed 12%of ischaemic oedema (submitted).

The main aim of this work was whether anti-allergic drugs, cromolycate and nedocromil, increase the oedema suppression by Dex, because nedocromil is reported to possess more anti-inflammatory effects than other anti-allergic drugs.[4] Nedocromil significantly inhibited the release of histamine, leukotriene $C_4$ ($LTC_4$) and prostaglandin $D_2$ ($PGD_2$) from bronchoalveolar cells in response to stimulation with antigen or antibody to human IgE. Cromoglycate had less than 1/200 the potency of nedocromil in these cells and failed to inhibit the antigen-induced bronchoconstriction in the *Ascaris*-sensitized primate *Macaca arctoides*. Nedocromil, but not cromoglycate, inhibited IgE-induced beta-hexosaminidase release from rat bone marrow-derived mast cells in spite of the fact that both drugs inhibited the release from peritoneal mast cells.[5] Although cromoglycate is used successfully in the treatment of asthma, it was recognized that the compound has limitations in the treatment of certain patients with intrinsic asthmatic and those in the older age group. Lately developed nedocromil was expected to

© 1997 Rapid Science Publishers cure these categories of patients as it possesses an anti-inflammatory character.[4] Nedocromil (1–10 μM) was also stronger as an anti-allergic drug than cromoglycate (10–100 μM) in *in vitro* tests. These drugs inhibited the anti-IgE-induced histamine release from bronchoalveolar lavage (BAL) cells of normal subject (12 males, age 22–74 years).[6] The anti-asthmatic action of cromoglycate has been supposed to affect sensory nerves which can influence the airway calibre, because this drug is neither atropine-like, nor anti-histamine and it does not relax the bronchial smooth muscle.[7] More potent suppression by nedocromil than cromoglycate was expected with Dex in our ischaemic and histamine paw oedema after these reports. Nevertheless, these drugs were equipotent to suppress our models, suggesting that they have still another common unknown capacity to enhance the oedema suppression which resembles that of a certain natural antioxidant. In this experiment, the enhancement of Dex action by cromoglycate or nedocromil was impaired by superoxide dismutase (SOD) and catalase, but not by a nitric oxide synthase (NOS) inhibitor. This suggests that the endogenous amount of superoxide radical ($O_2^-$) and/or hydrogen peroxide ($H_2O_2$) were required for Dex action which was enhanced by cromoglycate or nedocromil.

### Materials and Methods

### Animals and assay

Male ddY mice were obtained from SLC Co. (Shizuoka) and weighed 1–2 h before the experiment to select 30–36 g mice after keeping in room temperature for at least 1 day. The binding of the right hind paw with a commercial rubber ring ($1 \times 1$ mm, d = 42 mm) was served to induce ischaemia. Mice with various amounts of rubber binding (8–14 times) just above the articulation were examined with different duration of ischaemia (5–120 min) and duration of natural blood recirculation (5–180 min) after scissoring off the rubber. Ischaemic paw oedema provoked by 20 min ischaemia with 10 times of rubber binding followed by 20 min recirculation, was found to be the best for evaluating the suppression or enhancement by the drug. The rubber binding was performed under light ether anaesthesia which was permitted by the ethical guide line of the Japan Experimental Animal Association. Details of the manipulation using a plastic cylinder device was in our previous report.[8] The increase of paw thickness (not circumference) was obtained as a difference of thickness measured with Citizen Thickness gauge (Citizen Watch Co., Tokyo) before and after the ischaemic insult. The average increase of paw thickness in control animals was 0.77–0.83 mm ($n = 5$, 18 experiments). Assays were performed at 1, 3, 6 and 18 h after Dex. Histamine paw oedema was induced by 10 μl of saline solution containing net 3 μg histamine.[3] Paw swellings of control mice was 0.79–0.88 mm after 13 min when the paw swelled to maximum ($n = 5$, 15 experiments). Drug solution was prepared 1 to 3 h before injection (0.2 ml/10 g body weight, i.p.). Dex (0.2 ml/10 g body weight, s.c.) was injected 30 min after anti-allergic drug. Inhibitor (SOD etc., saline solution) was injected (0.2 ml/10 g body weight) just before anti-allergic drug. The increased paw thickness of drug- and/or inhibitor-treated mouse ($n = 3$) were compared with the average value of control ($n = 5$) in each experimental day for calculation of suppression. Tests for one dose were repeated four times on different days ($n = 12$ in total). Suppression was expressed as the mean ± standard error (SE) of the mean. Statistical significance was determined using Student's paired *t*-test.

### Drugs

Dexamethasone (Dex, Decadoron®, 4 mg/ml as phosphate ester) was purchased from Banyu Pharmaceutical Co. (Tokyo). Cu,Zn-superoxide dismutase (Cu,Zn-SOD, bovine erythrocyte, 3000 U/mg solid) and $N^G$-nitro-L-arginine methyl ester (L-NAME) were provided by Sigma Co. (St Louis, USA). Catalase (bovine liver, 1500 U/mg solid) was the product of Tokyo Kasei Chem. Co. (Tokyo). Disodium cromoglycate (DSCG, Intal®, called cromoglycate in this report) and disodium nedocromil (called nedocromil in this report) were synthesized in this laboratory for experimental use. Other chemicals including histamine ·2HCl, were analytical grade reagents obtained from Nakalai Tesk Chem. Co. (Kyoto).

### Results

Dexamethasone (Dex, 0.1 mg/kg, s.c.) suppressed neither ischaemic nor histamine paw oedema at 1 h after injection. Both 1 and 10 mg/kg Dex (s.c., i.p., i.v.) failed to suppress these oedemata at 1 h because Dex did not act directly and needed a lag time to induce anti-inflammatory protein(s). Maximum suppression by Dex alone was observed at 3 h in ischaemic paw oedema. Suppression of this oedema in 30 mg/kg cromoglycate- or nedocromil-treated mice, was not observed at 1 h, but significantly increased at 3, 6, 8 and 18 h after Dex (Fig. 1A). In histamine paw oedema, the enhancements of suppressions by these anti-allergic drugs were evident at 3–8 h after Dex (Fig. 1B).

As the prolonged suppressions by Dex plus the anti-allergic drugs seemed to be beneficial and important for clinical trial, the dose–response relations of these drugs were examined at 6 h after 0.1 mg/kg Dex. Significant increases in suppressions by Dex (0.1 mg/kg, s.c.) were found with 1–30 mg/kg cromoglycate or nedocromil in both models of paw oedemata (Fig. 2A,B). Cromoglycate suppressed ischaemic paw oedema even by 0.1 mg/kg at 6 h after Dex.

Suppression by 0.1 mg/kg Dex alone at 6 h was slightly enhanced by 30 mg/kg SOD and by 100 mg/kg L-NAME in ischaemic paw oedema. However, SOD, catalase and cycloheximide reversed the suppression by Dex plus cromoglycate. L-NAME did not change the cromoglycate-enhanced suppression by Dex (Fig. 3, left). Nedocromil-enhanced Dex suppression was also impaired by SOD, catalase and cycloheximide, but not by L-NAME. Similar impairments of Dex suppressions which were enhanced by cromoglycate or nedocromil, were also observed in histamine paw oedema (Fig. 3, right). Endogenous amount of $O_2^-$ and/or $H_2O_2$ seemed to be essential for the increase in suppressions by Dex plus an anti-allergic drug. Therefore, excess scavenging of these oxidants by a redox enzyme plus an anti-allergic drug, decreased Dex action in our paw oedema models. L-NAME alone suppressed both models and did not influence the suppressions of Dex that were enhanced by cromoglycate or nedocromil. This suggested that the role of endogenous NO was a little inhibitory against Dex suppression. Cycloheximide co-injection always impaired the Dex suppression, thus the protein synthesis was required for suppression by Dex regardless of additional administration of an anti-allergic drug.

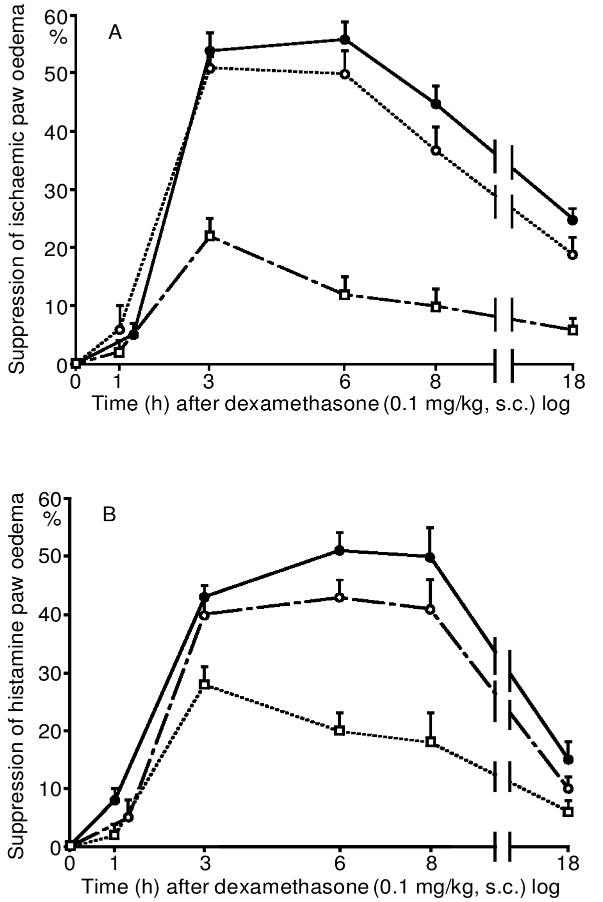

FIG. 1. Time-course of paw oedema suppression. (A) Ischaemic paw oedema. (B) Histamine paw oedema. □: Dex (0.1 mg/kg, s.c.) alone; ●: Dex plus cromoglycate (30 mg/kg, i.p.); ○: Dex plus nedocromil (30 mg/kg, i.p.). Drug was injected 30 min before Dex. Vertical lines represent standard error (SE) of means ($n = 12$).

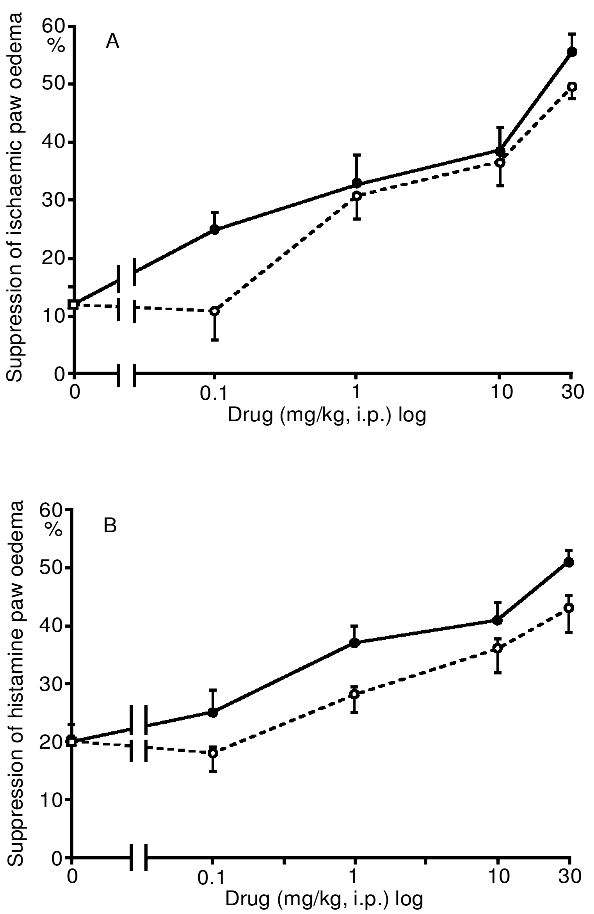

FIG. 2. Dose-response suppression of paw oedema at 6 h after Dex (0.1 mg/kg, s.c.). (A) Ischaemic oedema. (B) Histamine paw oedema. ●: Dex + cromoglycate, ○: Dex plus nedocromil. Drug was injected 30 min before Dex. Vertical lines represent SE of means ($n = 12$).

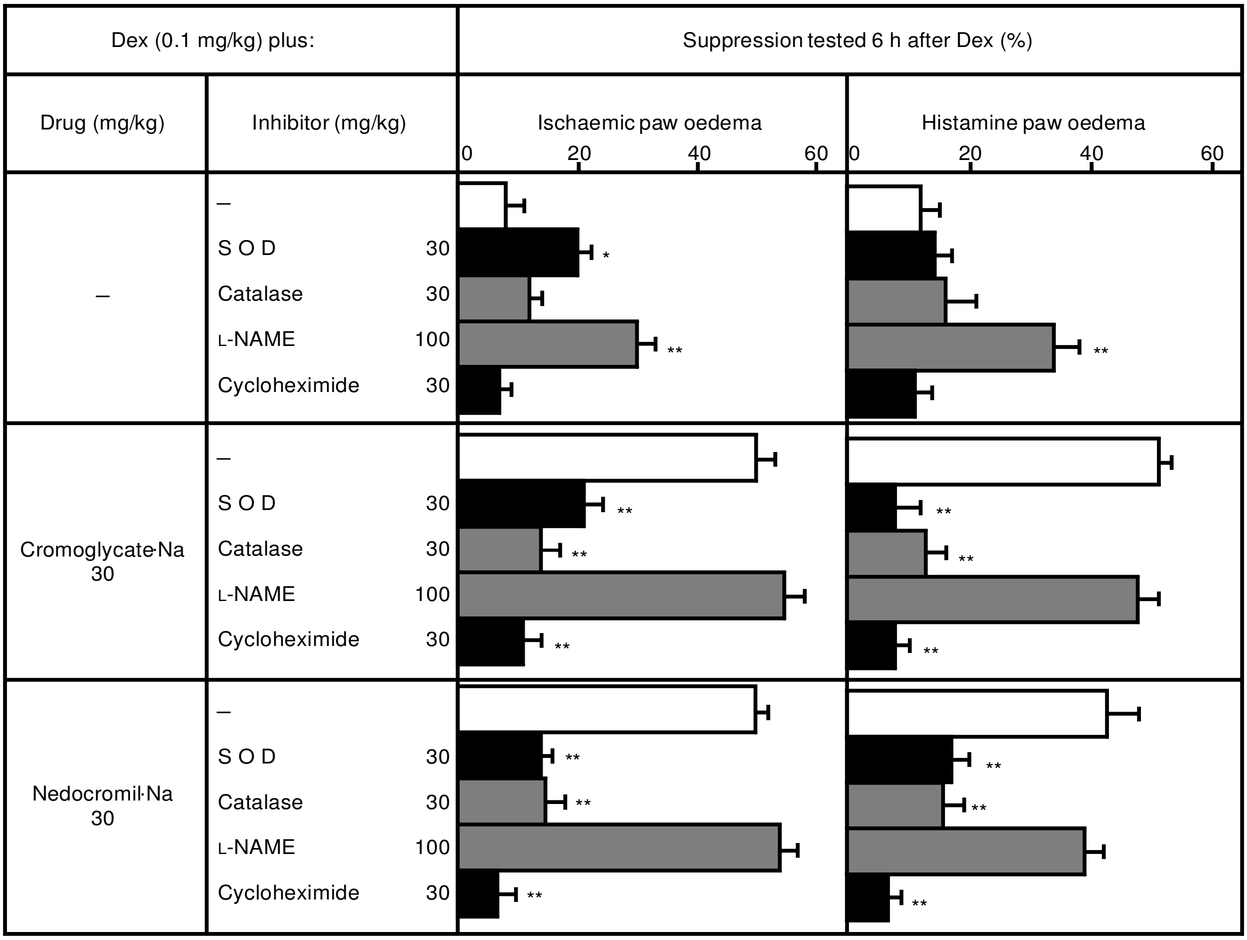

FIG. 3. Effect of inhibitor on oedema suppression by Dex alone and Dex + drug (cromoglycate or nedcromil). Tests were performed at 6 h after Dex (0.1 mg/kg, s.c.). Drug was injected i.p. 30 min before Dex. Administration of inhibitor (i.p.) was administered before drug. Vertical lines represent SE of mean by four times tests with three mice ($n = 12$, in total). ** and * shows statistical differences ($P < 0.01$ and $P < 0.05$ to each control, respectively). Control mice received Dex ± drug and no inhibitor.

### Discussion

Eosinophil chemotaxis and adherence were inhibited by $10^{-5}$ M nedocromil.[9] This anti-allergic drug was more potent than cromoglycate in inhibiting the release of histamine and leukotriene $C_4$ ($LTC_4$) in human mast cells. Nedocromil, but not cromoglycate, had a protective effect in canine models with airway $SO_2$ exposure and cough.[10] Nedocromil is thus believed to possess an anti-inflammatory character. Inhibition by nedocromil of superoxide radical ($O_2^-$) generation in human neutrophils[11] and of activation of mast cells, macrophages, eosinophils and epithelial cells[12] also showed this character. The results of a clinical double-bind, placebo-controlled comparison with salbutamol, showed that nedocromil equipotently decreased airway inflammation.[12] However, the mechanism of an anti-inflammatory action of nedocromil was different with FK506 or Dex. Sensitized and antigen-stimulated vehicle-treated guinea pigs showed marked infiltration of the bronchial wall by $CD4^+$ T-lymphocytes and eosinophils. FK506 and Dex abolished this infiltration but nedocromil failed to abolish it, therefore the anti-inflammatory effect of nedocromil was not associated with T-lymphocytes or eosinophils.[13]

In our experiments, cromoglycate and nedocromil alone were weak in suppressing the ischaemic and histamine paw oedema of mice (Fig. 2A,B), but they enhanced the suppression by a low dose (0.1 mg/kg) of Dex. Immunosuppressants such as FK506 failed to suppress these oedemata, but accelerated and increased the suppression by Dex at 3 h after Dex injection.[3] Suppressions of our paw oedemata decreased after 3 h of 0.1 mg/kg Dex. Cromoglycate and nedocromil (1–30 mg/kg, i.p.) prolong the effective duration time of Dex action up to 8 h (Fig. 1A,B) without inducing the early appearance of oedema suppression at

30 min or 1 h after Dex. Natural antioxidants (morin, α-tocopherol, tannic acid, rutin, bilirubin, β-carotene, quercetin) alone were ineffective in suppressing ischaemic and histamine paw oedema, but prolonged the suppression time by 0.1 mg/kg Dex (our unpublished data). In this context, the mode of anti-allergic drug suppression resembled those of natural antioxidants. The maintenance of a cellular reductive state in target cells by these antioxidants was supposed to be the cause for prolonging Dex action, for the glucocorticoid receptor (GR) complex might be more active in a reductive state to produce Dex-inducible proteins such as lipocortin[1] or vasoregulin.[2] After the mode of oedema suppression, cromoglycate and nedocromil must be similar to natural antioxidants thereby increasing Dex-inducible anti-inflammatory proteins.

Immunosuppressants including FK506 enhanced the oedema suppression by Dex in a different mode from two anti-allergic drugs and natural antioxidants. GR complex contains heat shock proteins (hsp)—hsp56 or cyclophilin-40 (CyP-40), hsp70, hsp90 and other unidentified low molecular hsps. FK506 and rapamycin bind to hsp56.[14] Cyclosporin A binds to CyP-40[15]. Deoxyspergualin binds to hsp70 and hsp90. These bindings of immunosuppressants might change the conformation of GR complex and thus facilitate the activation of inactive GR complex by dissociating two hsp90 molecules. The increased entrance of activated GR complex into the nucleus or enhanced attachment of GR complex to GRE (an enhancer site on DNA chain) is also possible by this conformational change. The possible site(s) to enhance the signalling of glucocorticoid (GC) such as Dex, was sensitive to a nitric oxide synthase (NOS) inhibitor, $N^G$-nitro-L-arginine methyl ester, Cu,Zn-superoxide dismutase (Cu,Zn-SOD, $O_2^-$ scavenger) and mannitol (hydroxyl radical [·OH] scavenger).[3] This is true for Dex suppression with or without additional drugs. Therefore, the endogenous amount of NO, $O_2^-$, $H_2O_2$ or ·OH seemed to be essential for Dex action. Enhancement of oedema suppression by Dex plus anti-allergic drug, was reversed by Cu,Zn-SOD and catalase, but not by L-NAME (Fig. 3). Endogenous amount of NO must not be involved in the enhancing action of cromoglycate and nedocromil on Dex action in ischaemic paw oedema. The enhancing action of natural antioxidant (morin, bilirubin and β-carotene), was also reversed by each 30 mg/kg (i.p.) of Cu,Zn-SOD and catalase, but not by L-NAME (unpublished data). Scavenging capacity of ROS or NOS inhibitory action of cromoglycate and nedocromil has not yet been demonstrated *in vitro*, but they might behave by increasing the intracellular reductive state just as natural antioxidants. Oxidants such as hydrogen peroxide ($H_2O_2$) activated a transcription factor immunoglobulin κ light-chain binding nuclear factor (NF-κB) and reduced the activity of activator protein (AP-1).[16] GR is a transcriptional factor, therefore it is not surprising that the GC action was supported by the endogenous amount of superoxide radical ($O_2^-$) or $H_2O_2$. The modification of the GR complex by two anti-allergic drugs seemed impossible from the mode of action on oedema models (the lack of increased suppression at 1 h after Dex injection). Cycloheximide, a protein synthesis inhibitor, always impaired the suppression by Dex whether Dex alone or with a drug, indicating that the synthesis of anti-inflammatory proteins is essential for Dex action.[17] Clinical trials of combined therapy with a diminished dose of GC and cromoglycate or nedocromil must be hopeful for asthma and some kind of inflammations expecting less undesirable side effects of these drugs.

In conclusion, cromoglycate and nedocromil should have a common mechanism to enhance the suppression by low dose of Dex in ischaemic- and histamine-induced inflammations in addition to their reported anti-allergic and anti-inflammatory actions. This suppresive mechanism seemed to be supported by endogenous reactive oxygen species. Our combination therapy is worthwhile of trial in the clinical field.

### References

1. Blackwell GJ, Carnuccio M, DiRosa M, *et al.* Macrocortin: a polypeptide causing the antiphospholipase effect of glucocorticoid. *Nature* 1980; **287**: 147–149.
2. Oyanagui Y, Suzuki S. Vasoregulin, a glucocorticoid-inducible vascular permeability inhibitory protein. *Agents and Actions* 1985; **17**: 270–277.
3. Oyanogui Y. Nitric oxide- and hydrogen peroxide-mediated gene expression by glucocorticoid and FK506 in histamine paw edema of mice. *Life Sci* 1994; **55**: PL177–PL185.
4. Eady RP. The pharmacology of nedocromil sodium. *Eur J Respir Dis* 1986; **69** (suppl 147): 112–119.
5. Okayama Y, Benyon RC, Rees PH, *et al.* Inhibition profiles of sodium cromoglycate and nedocromil sodium on mediator release from mast cells of human skin, lung, tonsil, adenoid and intestine. *Clin Exp Allergy* 1992; **22**: 401–409.
6. Leung KBP, Flint KC, Brostoff J. Comparison of nedocromil sodium and sodium cromoglycate on human lung mast cells obtained by broncho-alveolar lavage and by dispersion of lung fragments. *Eur J Respir Dis* 1986; **69** (suppl 147): 223–226.
7. Dixon M, Jackson DM, Richard IM. The action of sodium cromoglycate on 'C' fibre endings in the dog lung. *Br J Pharmac* 1980; **70**: 11–13.
8. Oyanagui Y, Sato S. Heparin, a potent releasing agent of extracellular superoxide dismutase (EC-SOD C), suppresses ischemic paw oedema in mice. *Free Rad Res Comm* 1990; **9**: 87–99.
9. Abdelaziz MM, Devalia JL, Khair OA, *et al.* The effect of nedocromil sodium on human airway epithelial cell-induced eosinophil chemotaxis and adherence to human endothelial cell in vitro. *Eur Respir J* 1997; **10**: 851–857.
10. Orr TSC. Nedocromil sodium: a new therapeutic option for reversible obstructive airway disease. *Br J Clin Pract* 1987; **41** (suppl 53): 9–11.
11. Rubin RP, Thompson RH, Naps MS. Differential inhibition by nedocromil sodium of superoxide generation elicited by platelet activating factor in human neutrophils. *Agents and Actions* 1990; **31**: 237–242.

12. Devalia JL, Rusznak C, Abdelaziz MM, *et al.* Nedocromil sodium and airway inflammation in vivo and in vitro. *J Allergy Clin Immunol* 1996; **98**: S51–S57.
13. Lapa e Silva JR, Ruffié C, Vargaftig, *et al.* Modulation of the bronchial inflammation in sensitized guinea-pigs by FK506, nedocromil sodium and dexamethasone. *Eur Respir J* 1995; **8**: 1321–1327.
14. Ning Y-M, Sanchez ER. Potentiation of glucocorticoid receptor-mediated gene expression by the immunophilin ligands FK506 and rapamycin. *J Biol Chem* 1993; **268**: 6073–6076.
15. Owens-Grillo JK, Hoffmann K, Hutchison KA, *et al.* The eyclosporin A-binding immunophilin CyP-40 and the FK506-binding immunophilin hsp56 bind to a common site on hsp90 and exist in independent cytosolic heterocomplexes with the untransformed glucocorticoid receptor. *J Biol Chem* 1995; **270**: 20479–20484.
16. Schenk H, Klein M, Erdbügger W, *et al.* Distinct effects of thioredoxin and antioxidant on the activation of transcription factor NF-κB and AP-1. *Proc Natl Acad Sci USA* 1994; **91**: 1672–1676.
17. Oyanagui Y. Immunosuppressants and TGF-β1 accelerated and prolonged the nitric oxide/oxyradicals-dependent suppression by dexamethasone in paw edema of mice. *Life Sci* 1996; **58**: PL287–PL294.

**Received 4 August 1997; accepted in revised form 29 August 1997**
